# Efficacy of HDAC Inhibitors Belinostat and Panobinostat against Cisplatin-Sensitive and Cisplatin-Resistant Testicular Germ Cell Tumors

**DOI:** 10.3390/cancers12102903

**Published:** 2020-10-10

**Authors:** João Lobo, Catarina Guimarães-Teixeira, Daniela Barros-Silva, Vera Miranda-Gonçalves, Vânia Camilo, Rita Guimarães, Mariana Cantante, Isaac Braga, Joaquina Maurício, Christoph Oing, Friedemann Honecker, Daniel Nettersheim, Leendert H. J. Looijenga, Rui Henrique, Carmen Jerónimo

**Affiliations:** 1 Cancer Biology and Epigenetics Group, IPO Porto Research Center (GEBC CI-IPOP), Portuguese Oncology Institute of Porto (IPO Porto) & Porto Comprehensive Cancer Center (P.CCC), R. Dr. António Bernardino de Almeida, 4200-072 Porto, Portugal; jpedro.lobo@ipoporto.min-saude.pt (J.L.); catarina.guimaraes.teixeira@ipoporto.min-saude.pt (C.G.-T.); daniela.silva@ipoporto.min-saude.pt (D.B.-S.); Vera.Miranda.Goncalves@ipoporto.min-saude.pt (V.M.-G.); vania.gomes.camilo@ipoporto.min-saude.pt (V.C.); rita.guimaraes@ipoporto.min-saude.pt (R.G.); marianacantantecf@gmail.com (M.C.); 2 Department of Pathology, Portuguese Oncology Institute of Porto (IPOP), R. Dr. António Bernardino de Almeida, 4200-072 Porto, Portugal; 3 Department of Pathology and Molecular Immunology, Institute of Biomedical Sciences Abel Salazar, University of Porto (ICBAS-UP), Rua Jorge Viterbo Ferreira 228, 4050-513 Porto, Portugal; 4 Princess Máxima Center for Pediatric Oncology, Heidelberglaan 25, 3584 CS Utrecht, The Netherlands; L.Looijenga@prinsesmaximacentrum.nl; 5 Department of Urology, Portuguese Oncology Institute of Porto (IPOP), R. Dr. António Bernardino de Almeida, 4200-072 Porto, Portugal; isaac.braga@ipoporto.min-saude.pt; 6 Department of Medical Oncology, Portuguese Oncology Institute of Porto (IPOP), R. Dr. António Bernardino de Almeida, 4200-072 Porto, Portugal; jmauricio@ipoporto.min-saude.pt; 7 Department of Oncology, Hematology and Bone Marrow Transplantation with Section of Pneumology, University Medical Center Hamburg-Eppendorf, Martinistraße 52, 20246 Hamburg, Germany; c.oing@uke.de (C.O.); Friedemann.Honecker@zetup.ch (F.H.); 8 Laboratory of Radiation Biology and Experimental Radiation Oncology, University Medical Centre Hamburg-Eppendorf, 20246 Hamburg, Germany; 9 Tumour and Breast Center ZeTuP St. Gallen, Rorschacher Strasse 150, 9006 St. Gallen, Switzerland; 10 Department of Urology, Urological Research Lab, Translational UroOncology, University Hospital Düsseldorf, 40225 Düsseldorf, Germany; Daniel.Nettersheim@med.uni-duesseldorf.de

**Keywords:** testicular germ cell tumors, cisplatin resistance, targeted therapies, epidrugs, HDAC inhibitors, belinostat, panobinostat

## Abstract

**Simple Summary:**

There is a need for novel treatment options for patients with testicular germ cell tumors, especially for those that are resistant to standard chemotherapy, who show poor prognosis. In this work, we test two compounds that inhibit epigenetic enzymes called histone deacetylases—belinostat and panobinostat. We show that these enzymes are expressed at different levels in different germ cell tumor subtypes (seminomas and non-seminomas) and that both drugs are effective in reducing tumor cell viability, by decreasing cell proliferation and increasing cell death. These results are promising and should prompt further works with these compounds, envisioning the improvement of care of germ cell tumor patients.

**Abstract:**

Novel treatment options are needed for testicular germ cell tumor (TGCT) patients, particularly important for those showing or developing cisplatin resistance, the major cause of cancer-related deaths. As TGCTs pathobiology is highly related to epigenetic (de)regulation, epidrugs are potentially effective therapies. Hence, we sought to explore, for the first time, the effect of the two most recently FDA-approved HDAC inhibitors (HDACis), belinostat and panobinostat, in (T)GCT cell lines including those resistant to cisplatin. In silico results were validated in 261 patient samples and differential expression of HDACs was also observed across cell lines. Belinostat and panobinostat reduced cell viability in both cisplatin-sensitive cells (NCCIT-P, 2102Ep-P, and NT2-P) and, importantly, also in matched cisplatin-resistant subclones (NCCIT-R, 2102Ep-R, and NT2-R), with IC50s in the low nanomolar range for all cell lines. Treatment of NCCIT-R with both drugs increased acetylation, induced cell cycle arrest, reduced proliferation, decreased Ki67 index, and increased p21, while increasing cell death by apoptosis, with upregulation of cleaved caspase 3. These findings support the effectiveness of HDACis for treating TGCT patients in general, including those developing cisplatin resistance. Future studies should explore them as single or combination agents.

## 1. Introduction

Testicular germ cell tumors (TGCTs) are among the most common solid cancers in young-adult Caucasian men, and the incidence is rising due to several environmental factors. These tumors are closely related to developmental biology, which constitutes the basis of their classification [1,2]. Among these, type II TGCTs are the most common and the most clinically challenging, due to malignant behavior. They are histologically classified into seminomas (SEs) or the heterogeneous family of non-seminomas (NSs), the latter including embryonal carcinoma (EC), choriocarcinoma (CH), yolk sac tumor (YST), and teratoma (TE) subtypes, as well as mixed tumors (comprising mixtures of several components) [3]. An accurate histological characterization is key since it has implications in clinical course and therapy selection [4].

Overall, the prognosis of the disease is good, with five-year overall-survival rates over 95%, and most patients are effectively treated even in the event of metastasized disease, which is in great part due to the high sensitivity of these tumors to platin-based chemotherapy [5,6]. However, major clinical challenges in the field persist. Firstly, there is a risk of overtreatment of patients that would never experience recurrence and might be spared the long-term side effects of chemotherapy [7]. Hence, there is a need for biomarkers that identify patients that can safely avoid adjuvant chemotherapy following surgical resection, and also for alternative, less-toxic drugs to efficiently treat such young patients. Secondly, there is a proportion of patients displaying/developing cisplatin resistance, eventually succumbing to the disease in a few months [8]; again, novel biomarkers are needed to identify upfront which patients will have or develop resistance and, very importantly, there is an urgent need for new therapies for these patients since no validated effective treatment options are available [9].

Despite intense research efforts directed towards a better understanding of cisplatin sensitivity and resistance in this tumor model, a major unifying mechanism is still lacking which may be therapeutically targeted [10,11,12,13]. Most likely, the biology of cisplatin resistance is multifactorial [12,14]. Importantly, epigenetic (de)regulation has been implicated as contributing to resistance by interfering with several candidate mechanisms and pathways [15,16,17,18]. Hence, we hypothesize that epidrugs may be a broad and effective way of treating cisplatin-resistant TGCTs [19,20]. Indeed, past studies have shown promising results for demethylating agents, as well as histone deacetylase inhibitors (HDACis) and bromodomain inhibitors [21,22,23,24,25,26,27].

Herein, we aimed to assess the therapeutic potential of the two most recently Food and Drug Administration (FDA)-approved HDACis, belinostat and panobinostat, in (T)GCT cell lines including those resistant to cisplatin. We show that both agents, at low nanomolar concentrations, effectively reduce cell viability, and reduce the proliferation of cell lines, whereas apoptosis was increased.

## 2. Results

### 2.1. HDACs Are Differentially Expressed among TGCT Patient Samples, Including Those Exposed to Cisplatin: In Silico Analyses and Validation

**Figure 1:**
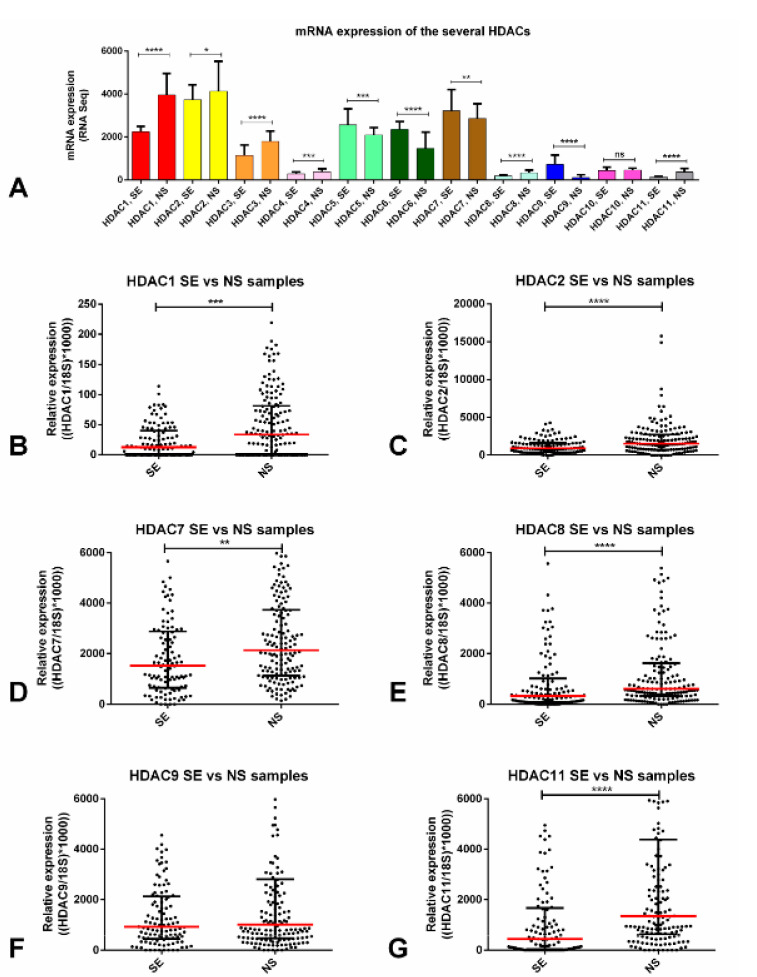
Differential mRNA expression of several HDAC isoforms. (**A**) mRNA expression of the several HDAC isoforms across the TCGA database—in silico analysis; transcript levels of several HDACs—validation in our patient cohort: (**B**) HDAC1, (**C**) HDAC2, (**D**) HDAC7, (**E**) HDAC8, (**F**) HDAC9, (**G**) HDAC11. Bars and red dashes represent the median and interquartile range. Abbreviations: HDAC—histone deacetylase; SE—seminoma; NS—non-seminoma.* *p* < 0.05; ** *p* < 0.01; *** *p* < 0.001, **** *p* < 0.0001.

We first explored the expression of the several HDACs among the 156 TGCT tumor samples of the TCGA database. For the purposes of this study, we focused on the 11 Zn^2+^ -dependent HDACs (class I, II, and IV, targeted by belinostat and panobinostat), disregarding the sirtuins’ family (class III), which is NAD^+^ dependent. We found that class I HDAC1 and HDAC2 were expressed at higher levels when compared to the remaining players. Also, most HDACs (HDACs 1, 2, 3, 4, 8, and 11) were upregulated in NSs compared to SEs. HDACs class II (like HDAC 5, 6, 7, and 9), on the other hand, were upregulated in SEs compared to NSs (Figure 1A).

Next, we selected HDACs (at least one from each class) that were expressed at high levels overall or, importantly, we focused on those in which differential expression between SEs and NSs was significant (i.e., those with upregulation in NS, since the cell lines used in our work were representative of NS). Using real-time quantitative polymerase chain reaction (RT-qPCR), HDAC2 disclosed the highest expression levels. We also verified that HDAC1, 2, 7, 8, and 11 were significantly upregulated in NS tumor samples compared to SEs (Figure 1B–G). When discriminating each tumor subtype, the SE components were the ones with significantly lower expression of HDACs, except for HDAC9 (Figure S1A–F). No significant associations with clinicopathological features were found, except for HDAC7 upregulation in stage III disease (*p* = 0.0072) (Figure S1G).

**Figure 2:**
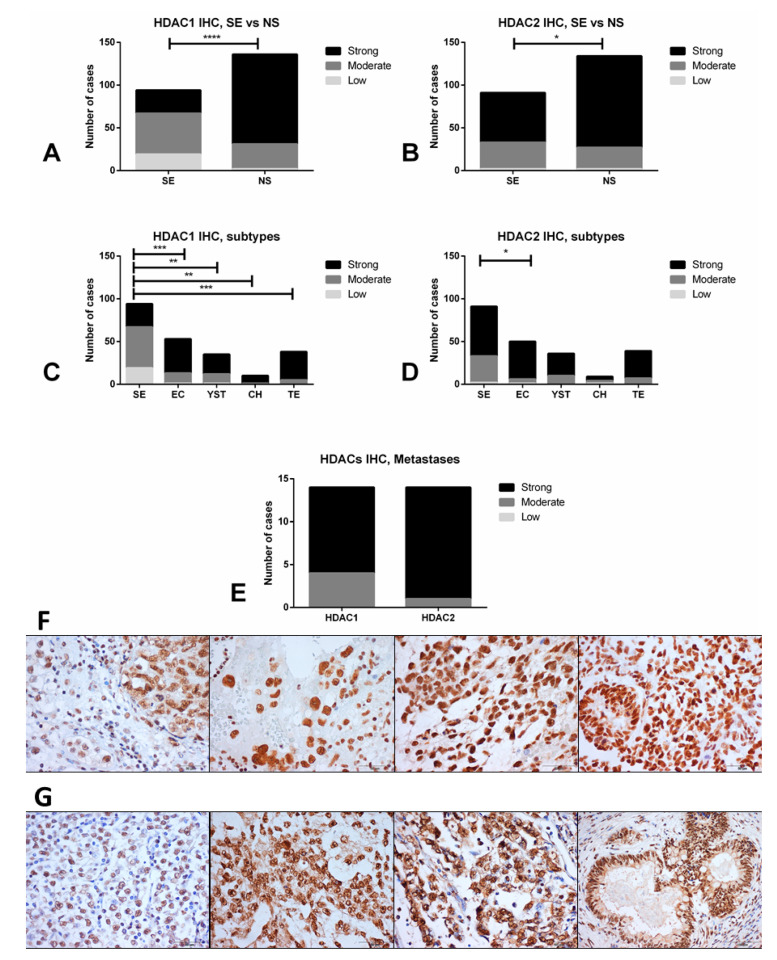
Differential immunoexpression of HDAC1 and HDAC2 in TGCT samples. (**A**,**B**) comparison between seminoma and non-seminoma; (**C**,**D**) comparison across all TGCT subtypes; (**E**) immunoexpression of HDAC1 and 2 in cisplatin-exposed metastatic samples; (**F**) illustrative examples of immunoexpression of HDAC1 (left to right: mixed tumor composed of seminoma and embryonal carcinoma, choriocarcinoma, yolk sac tumor, immature teratoma); (**G**) illustrative examples of immunoexpression of HDAC2 (left to right: seminoma, yolk sac tumor, embryonal carcinoma, mature teratoma). Abbreviations: HDAC—histone deacetylase; SE—seminoma; NS—non-seminoma; YST—yolk sac tumor; CH—choriocarcinoma; TE—teratoma; EC—embryonal carcinoma. * *p* < 0.05; ** *p* < 0.01; *** *p* < 0.001, **** *p* < 0.0001, Scale bars are indicated in each figure indicating the magnification.

Finally, we selected HDAC1 (reported to be the main HDAC used by (T)GCT cell lines for deacetylation [26]) and HDAC2 (for being the highest expressed in our tissue cohort) for validation at the protein level by immunohistochemistry. Indeed, we confirmed that NS samples depicted higher immunoexpression intensity scores compared to SE samples for HDAC1 (*p* < 0.0001) and HDAC2 (*p* = 0.0268) (Figure 2A–D). The highest immunoexpression scores were disclosed for HDAC2, with 73.3% of tumors showing strong immunoexpression, in line with the RNA based analyses. Importantly, immunoexpression of these HDACs was particularly high in cisplatin-exposed metastatic samples, with 10/14 (71.4%) and 13/14 (92.9%) tumors with strong immunoexpression of HDAC1 and HDAC2, respectively (Figure 2E, including 4/6 and 6/6 of non-teratoma cases, specifically). Illustrative examples of immunoexpression of these HDACs is depicted in Figure 2F,G.

Our data demonstrate that HDACs are differentially expressed among TGCT subtypes, being upregulated in NSs and show high protein expression in those exposed to cisplatin.

### 2.2. HDACs Are Differentially Expressed among (T)GCT Cell Lines, Including the Resistant and Parental Subclones

Next, we explored the expression of the previously selected HDACs in four (T)GCT cell lines. HDAC1 and HDAC2, followed by HDAC7′s transcript levels were the highest and significantly differed among the various cell lines. Overall, the SE-like TCam-2 cell line showed the lowest expression levels for most HDACs, except for HDAC7, where it was upregulated compared to other cell lines. The highest expression of HDAC1, 2, 8, 9, and 11 was observed in NCCIT or 2102Ep cell lines (Figure S2A–F). At the protein level, using Western blot and band quantification, we confirmed that class I HDACs (HDAC1, 2, and 8) were indeed expressed at lower levels in the TCam-2 cell line (Figure S2G–I).

**Figure 3:**
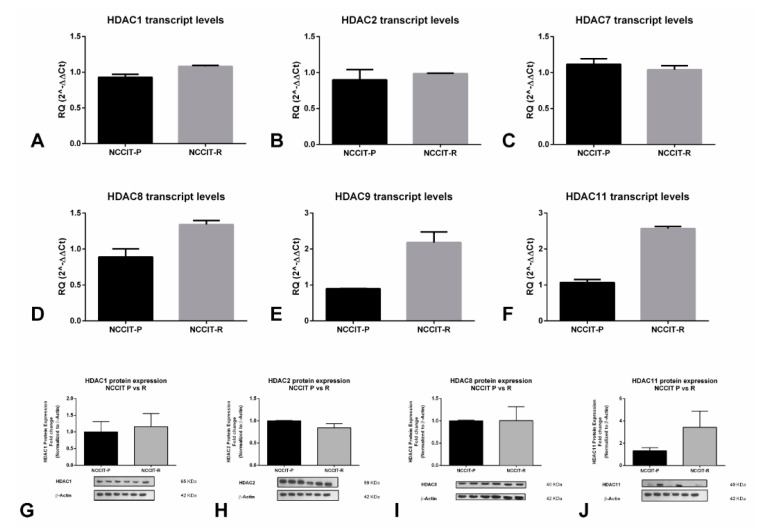
Differential expression of HDAC isoforms in cisplatin-sensitive vs cisplatin-resistant clones of the NCCIT cell line. Differential mRNA expression of (**A**) HDAC1, (**B**) HDAC2, (**C**) HDAC7, (**D**) HDAC8, (**E**) HDAC9, and (**F**) HDAC11; differential protein expression of (**G**) HDAC1, (**H**) HDAC2, (**I**) HDAC8, and (**J**) HDAC11. Abbreviations: HDAC—histone deacetylase; RQ—relative quantity.

We also explored the expression of selected HDACs on the cell line showing the highest resistance to cisplatin (NCCIT-R, see Section 2.3) and its matched parental clone NCCIT-P. We found that HDAC8, 9, and 11 mRNA expression were higher in the resistant clone compared to the parental clone, whereas expression of HDAC1, 2 and 7 remained unchanged (Figure 3A–F). We confirmed higher HDAC11 protein expression in NCCIT-R compared to NCCIT-P, reflecting transcript findings, although no remarkable differences were found for HDAC1, 2, and 8 (Figure 3G–J).

### 2.3. Validation of Cisplatin Resistance in the (T)GCT Cell Lines

To prove the induced resistance to cisplatin in the cell lines used in our study, the matched resistant and sensitive clones of the cell lines were treated with several concentrations of cisplatin for 72 h, as previously reported [28]. The IC50 of cisplatin for NCCIT-P was 2.92 µM, 10-times lower compared to that of NCCIT-R (31.59 µM); for 2102Ep-P it was 3.26 µM, four-times lower compared to that of 2102Ep-R (15.61 µM); for NT2-P it was 0.92 µM, three-times lower compared to that of NT2-R (3.31 µM). NCCIT-R was the most cisplatin-resistant cell line, followed by 2102Ep-R and finally NT2-R (Figure S3).

### 2.4. Treatment of Cisplatin-Sensitive and Cisplatin-Resistant Cell Lines with Belinostat or Panobinostat Decreases Cell Viability

**Figure 4:**
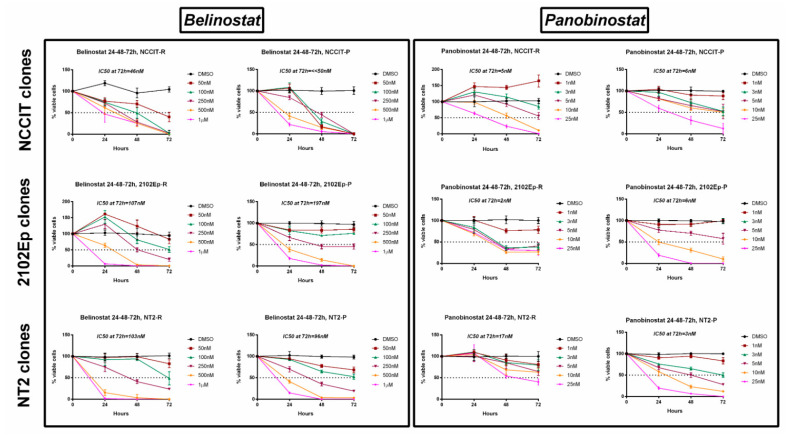
Viability curves across 72 h of treatment of the several cisplatin-sensitive and cisplatin-resistant (T)GCT cell lines with belinostat and panobinostat. Experiments were done in triplicates, normalized to DMSO, and normalized to day zero. IC50s at 72 h of treatment are indicated on top of each graph. Abbreviations: DMSO—dimethyl sulfoxide.

We then treated resistant cell lines with different concentrations of belinostat (50 nM to 1 µM) or panobinostat (1 to 25 nM) for 72 h. A dose-dependent and time-dependent effect on cell viability (both cisplatin-sensitive and -resistant clones) was observed for both drugs and for all cell lines (Figure 4). Regarding the resistant clones, the IC50 for belinostat was 46, 107, and 103 nM for NCCIT-R, 2102Ep-R, and NT2-R, respectively; panobinostat was even more effective, with an IC50 of only 5, 2, and 17 nM for the same cell lines (a full graphical representation of cell viability per time point and respective statistical analysis is illustrated in Figure S4). The same was verified in the cisplatin-sensitive clones, with IC50 for belinostat of 197 and 96 nM in 2102Ep-P and NT2-P (and absence of viable cells with the lowest concentration of 50 nM for NCCIT-P) and IC50 for panobinostat of 6, 6, and 3 nM for NCCIT-P, 2102Ep-P, and NT2-P, respectively (a full graphical representation of cell viability per time point and respective statistical analysis is illustrated in Figure S5).

Our data show that both HDACis are effective in reducing cell viability at low nanomolar concentrations, in three cisplatin-sensitive cell lines and, more importantly, also in the three matched cell line clones made resistant to cisplatin.

### 2.5. Treatment of Cisplatin-Resistant Cell Line NCCIT-R with Belinostat or Panobinostat Induces Cell Cycle Arrest and Promotes Apoptosis by Targeting Related Signaling Pathways, and Increases Acetylation Levels

Subsequently, we aimed to assess the impact on cell proliferation and apoptosis. For this, we chose the NCCIT-R cell line as surrogate, for being the most resistant (the highest IC50) to cisplatin.

**Figure 5:**
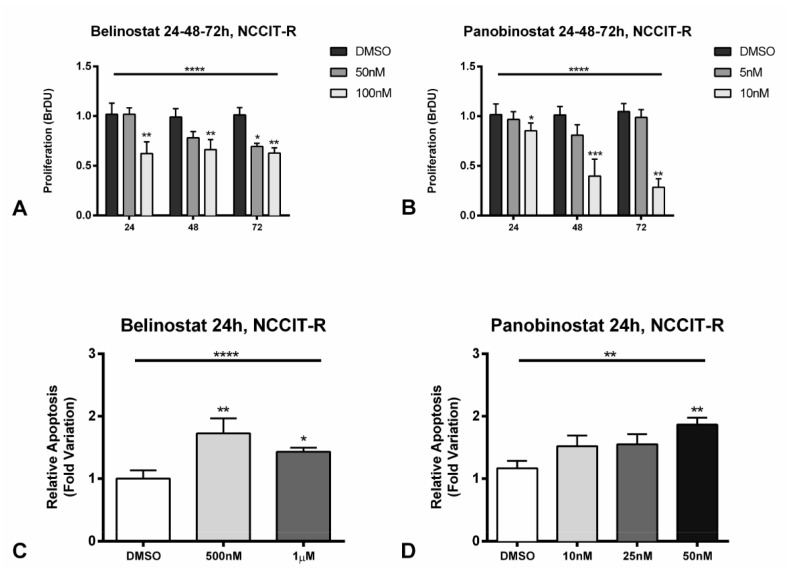
Effect on cell proliferation (**A**,**B**) and apoptosis (**C**,**D**) after treatment of cisplatin-resistant NCCIT-R cell line with belinostat and panobinostat. (**A**,**B**) The decrease in cell proliferation determined by BrDU assay at 24, 48, and 72 h of treatment with belinostat and panobinostat; (**C**,**D**) increase in apoptosis as determined by Cell-APOPercentage at 24 h of treatment with belinostat and panobinostat. Abbreviations: DMSO—dimethyl sulfoxide. * *p* < 0.05; ** *p* < 0.01; *** *p* < 0.001, **** *p* < 0.0001.

When treating NCCIT-R with different concentrations of belinostat (50 and 100 nM) and panobinostat (5 and 10 nM), corresponding approximately to the IC50 and 2 × IC50 for this cell line, a significant (*p* < 0.0001) decrease in cell proliferation was found comparing to the vehicle, assessed by BrDU assay (Figure 5A,B, including statistical significance for each individual condition). The decrease was again time- and dose-dependent, being greater for 100 and 10 nM of belinostat and panobinostat, respectively, and at 72 h of exposure. Accordingly, a decrease in Ki67 staining index (by immunofluorescence) was observed in cells with the same concentrations (Figure S6A). Finally, we also found an increase in p21 protein levels at 24 h of treatment with either HDACis, with only a slight decrease of p53 expression (Figure S6B).

Moreover, we found an increase in apoptosis already at 24 h for belinostat (*p* < 0.0001) and panobinostat (*p* = 0.0084) when using a specific apoptosis assay (Figure 5C,D). This was further validated for panobinostat at 24 h, with an increase in cleaved caspase 3 expression (Figure S6B).

Finally, we demonstrated that treatment with belinostat and panobinostat at 24 h led to a remarkable increase in lysine acetylation levels, increased acetylation of histone H3, and decreased HDAC1 protein expression (Figure S6B).

These data support the HDACis effect, promoting acetylation levels and inducing cell cycle arrest by increasing p21 and increasing cell death by apoptosis by increasing levels of cleaved caspase 3.

### 2.6. Pre-Treatment of Cisplatin-Resistant Cell Line NCCIT-R with Non-Toxic Concentrations of Belinostat Increases Sensitivity to Cisplatin

As belinostat was shown to increase decitabine’s effect in rescuing sensitivity to cisplatin in a cisplatin-resistant ovarian cancer cell line [29], we pre-treated NCCIT-R cells with non-toxic concentrations of belinostat for 72 h and exposed treated cells to 10 µM cisplatin, which was shown to have no effect on NCCIT-R cell viability in the previous experiments. We observed that at 5, 10, and 20 nM concentrations of belinostat, an effect on cell viability was, indeed, not apparent (on the contrary, cells were actually proliferating), but subsequent treatment with cisplatin 10 µM considerably reduced cell viability, compared to the absence of effect when the same dose of cisplatin was given in the absence of belinostat pre-treatment (illustration of protocol and graphical representation in Figure S7).

These data suggest that HDACi pre-treatment in lower, non-toxic doses attenuates the mechanisms contributing to cisplatin resistance.

## 3. Discussion

Cisplatin resistance is the major cause of mortality and morbidity among TGCT patients, constituting a major clinical challenge [8,9]. Hence, novel treatments that efficiently target the cisplatin-resistant phenotype or that rescue sensitivity to platinum drugs are needed [30,31]. Such treatments would have a significant impact in the field and, thus, clinical studies/trials, focusing on multiple relapsed and resistant patients have been carried out [9,32,33,34,35,36]. However, no agent has yet made it into the clinic. Given the influence of epigenetic deregulation in these tumors and the demonstrated influence of epigenetic mechanisms on the acquisition of cisplatin resistance, there is a rationale to hypothesize that epidrugs may be key for treating those patients, either as single agents or in combination [19]. Most pre-clinical (and also some clinical) studies have focused on demethylating agents, like 5-azacytidine, guadecitabine, and decitabine [22,23,24,37,38], with the first studies already reported in the 1990s [39]. In this study, we focused on HDACis, instead.

HDACs are involved in deacetylation of histone (and non-histone) proteins, usually associated with transcription repression, and are grouped into four classes. Changes in the expression of these enzymes have been reported in several cancers and, importantly, inhibitors have been used effectively as cancer treatment [40], including repurposed drugs (e.g., antifungal trichostatin A (TSA)) or drugs used for treating epilepsy (e.g., valproic acid or carbamazepine) [41], as well as the more recently developed inhibitors, some already approved by the FDA and others on clinical trial [42]. Overall, only a few studies assessed HDACs expression in detail. Omisanjo et al. [43], first reported positive immunostaining for HDAC1 in TGCTs (EC, SE, and TE), although only 32 tumor samples were tested. Meanwhile, Fritzsche et al. [44] assessed HDAC1, 2, and 3 immunoexpression and found these enzymes to be consistently expressed, with HDAC1 expressed at lower levels compared to the remaining two isoforms; this matches our tissue data, as HDAC2 was expressed at higher levels (both at mRNA and protein levels) compared to HDAC1 (Figure 1B,C and Figure 2A,B) in NSs predominantly. Moreover, those authors also reported significant differential expression of these enzymes between SEs and NSs, with high expression of HDAC1 in CHs, in accordance with our data which disclosed upregulation in NS subtypes (with 9/10 CHs showing strong HDAC1 immunoexpression, indeed, Figure 1 and Figure 2). Those authors also showed that the expression of these players did not associate with clinicopathological features, which parallels our observations (except for the upregulation of HDAC7 in stage III disease, Figure S1G). Our data on 261 TGCT tumor components, together with the in silico analysis of the TCGA database (156 samples) [45] constitutes the largest series reported thus far in which HDACs expression was analyzed, and confirms the differential expression of members of the various HDAC families among TGCT subtypes, indicating a distinctive role in the biology of SEs and, particularly, of NSs, in which most isoforms are upregulated. This is further supported by our in vitro data, with differential expression of HDACs at the mRNA and protein level, namely higher protein expression again in the NS cell lines compared to TCam-2 (Figure S2). HDAC1 was shown to be the most expressed HDAC isoform in these cell lines [26], in accordance with our data depicting the highest transcript levels for this isoform (Figure S2A). Moreover, Yin et al. [46] also support this mechanistically, emphasizing the role of HDAC1 (targeted by LSD1 and by means of modulating H4K16 acetylation) in maintaining pluripotency of EC cells. Additionally, our findings expand these previous studies by analyzing HDACs immunoexpression in cisplatin-exposed tissue samples. The high-intensity staining in a large proportion of these tumor samples (71.4% for HDAC1 and 92.9% for HDAC2, Figure 2E) suggests that HDACs are also relevant in the context of cisplatin-resistant phenotype, thus indicating that targeting with HDACis may be an effective therapy. The same is again supported by our in vitro results, disclosing an upregulation of some HDAC isoforms in NCCIT-R, compared to NCCIT-P, both at the mRNA and protein level (Figure 3). The differential expression of HDAC11 is particularly interesting and deserves to be explored in further studies, given the recently reported effect of HDAC11-specific inhibitors in eliminating treatment-resistant lung adenocarcinoma cells by targeting SOX2 [47], which is amplified in the NCCIT cell line [48].

Few works have focused on HDACis for treating (T)GCT cell lines. Nettersheim et al. [27] first showed that Romidepsin induced apoptosis in TCam-2 cells at concentrations >10 nM, and later treated the various (T)GCT cell lines with TSA, vorinostat, valproic, acid and romidepsin, demonstrating that these agents induced the expression of *PRAME* in cell lines representative of NS [49]. More recently, the same authors have focused on dissecting the molecular mechanisms of action of romidepsin [26,50], which involves DHRS2 and also the ARID1A-GADD45B-DUSP1 signaling pathway. Importantly, they demonstrated that romidepsin reduced cell viability across cell lines (including those that were cisplatin-resistant) in a time- and dose-dependent manner, starting at concentrations ≥ 5 nM [26]. These studies are supportive of the therapeutic benefit of HDAC inhibition for treating TGCTs. Several studies have also reported, however, on the side effects of romidepsin treatment, namely hematological and cardiac [51]. For all this, we focused on the two most recently FDA-approved HDACis (belinostat, approved in 2014 for treatment of peripheral T-cell lymphoma, and panobinostat, approved in 2015 for treatment of multiple myeloma [42]), reporting their effect on (T)GCT cell lines. We have made use of proven cisplatin-resistant clones of NS cell lines (confirmed by us, by exposing cells to cisplatin, and comparing with the matched sensitive clones, Figure S3), and disclosed that both drugs effectively reduced cell viability in a time- and dose-dependent manner, at low nanomolar concentrations (Figure 4). Panobinostat was the most effective drug, with the lowest IC50s. For NCCIT-R, only the 1 nM concentration did not reduce viability, whereas in 2102Ep-R even this concentration resulted in reduced cell viability at 72 h of treatment. The similar effect on distinct cell lines, with different p53 status (mutated for NCCIT and wild-type for the remainder), suggests that the mechanism is independent of p53. Additionally, both drugs were also effective in reducing the viability of matched parental clones. This suggests that those agents are effective for treating both cisplatin-sensitive and cisplatin-resistant tumors, and the fact that they were effective across the distinct cell lines indicates that they might be useful in the clinical context as well.

Moreover, we showed a significant increase in apoptosis and a decrease in cell proliferation when treating the cell line most resistant to cisplatin (NCCIT-R) with belinostat and panobinostat (Figure 5). We further validated these data by showing a decrease in proliferation index (Ki67, Figure S6A), and an increase in p21 and cleaved caspase 3 protein levels (Figure S6B). Indeed, apoptosis induction and cell cycle arrest are among the most frequently reported pathways elicited by HDACis [42], and these data are also in line with the results of Nettersheim et al. [26], which showed a decrease/maintenance in p53 levels and increase in p21, implying p21 induction in a p53-independent manner, contributing to G2/M-phase arrest [52]. Moreover, we demonstrated the effect of the drugs, by showing increased lysine acetylation and H3 acetylation, accompanied by a decrease in HDAC1 protein expression (Figure S6B).

Finally, following the work of Steele et al. [29], who showed that belinostat potentiated the effect of decitabine in rescuing sensitivity to cisplatin in a cisplatin-resistant ovarian cancer cell line, we demonstrated that a 72-h pre-treatment of NCCIT-R cells with 5 to 20 nM of belinostat promoted sensitivity to cisplatin, leading to reduced cell viability, compared to cisplatin exposure alone (Figure S7). This should be validated in future studies more dedicated to this specific question, since it may indicate that HDACis might also target mechanisms involved in the acquisition of cisplatin resistance. This may be useful in combination therapy, as demonstrated for the combination of romidepsin or belinostat with cisplatin and etoposide in lung cancer [53]. Indeed, the data of Kim et al. indicate that by inducing relaxation of the chromatin structure, these agents facilitate the action of DNA damaging agents, which could be useful for treating intrinsically resistant tumors [54].

In future works, we intend to pursue in vivo studies, to assess more closely the effectiveness of these agents and, importantly, their toxicity profile on control subjects. This will be fundamental to assess the practicability of implementing these drugs in the clinical setting. Moreover, we intend to validate these findings and to explore in more detail if and how HDACs may be involved in cisplatin-resistance, through the knock-down of specific HDAC isoforms using CRISPR-Cas9 technology. Of particular interest are the findings of Cipro et al. in neuroblastoma cell lines, who indicate that HDACis overcome hypoxia-induced cisplatin resistance by interfering with acetylation of HIF-1α [55,56], suggesting that the crosstalk between epigenetics and tumor metabolism may be an interesting pathway to explore [57]. Also, as in our work, it has been shown that activation of apoptosis occurs through the caspase 3-dependent pathway [55], and can be facilitated through both the p53-dependent and p53-independent pathways [54]. Despite our data supporting the p53-independent mechanism, it is worth exploring p53 downstream targets, including NOXA and PUMA [58], and other family members (p63/p73), as these can be epigenetically regulated (including restored expression of p63 in TGCT cell lines) [59,60]. Finally, as mentioned above, we intend to explore in-depth the pre-treatment with HDACis followed by cisplatin exposure and the simultaneous combination of both drugs, as a schedule-dependent synergy has been reported, which triggered increased PARP degradation and increased γH2AX phosphorylation [53].

## 4. Materials and Methods

### 4.1. In Silico Analysis

To explore the expression of the several HDACs among TGCT subtypes and select the most promising candidates for validation in our cohort, we explored The Cancer Genome Atlas (TCGA) database using the publicly available cBioPortal tool [61]. RNA sequencing data for the several HDACs was obtained from the “Testicular Germ Cell Cancer—TCGA, Firehouse Legacy” cohort of 156 samples as raw data and plotted/analyzed in Microsoft Excel 2016 (Microsoft, Redmond, WA, USA) and GraphPad Prism 6 (GraphPad Software, La Jolla, CA, USA).

### 4.2. Patient Samples

Patient tumor samples were retrospectively selected from type II TGCT patients undergoing radical inguinal orchiectomy between 2005 and 2017 at the Portuguese Oncology Institute of Porto (IPO Porto), Portugal. A total of 161 TGCT patients were included. Additionally, 14 metastatic samples exposed to cisplatin (including eight residual mature teratomas—intrinsically resistant—and six cisplatin-resistant non-teratoma tumor samples) were included. All patients were treated at IPO Porto by the same multidisciplinary team. Specimens were routinely fixed in formalin and embedded in paraffin (FFPE) for subsequent staining with Hematoxylin and Eosin (H&E) and histological examination. All histological material was reviewed by the same TGCT-dedicated pathologist according to the most recent 2016 World Health Organization classification (cohort full characteristics reported in [3]). Clinical charts were also reviewed, and patients staged according to the most recent American Joint Committee on Cancer (AJCC) 8th edition. Patients presenting with metastases at diagnosis were further categorized following the International Germ Cell Cancer Collaborative Group (IGCCCG) prognostic system [62]. Follow-up was last updated in May 2019.

From each patient, a representative tumor block (with all representative histological elements available), with >70% tumor cellularity and low necrosis content, was selected. Importantly, distinct tumor components within the 62 mixed tumors were individually dissected and independently considered for downstream analyses, allowing to conclude on expression patterns among histological subtypes (as previously reported by us [63]). Thus, a total of 261 individual tumor samples were analyzed and 8 and 3 μm-thick sections were ordered for RNA extraction and for immunohistochemistry, respectively.

A summary of the study cohort is depicted in Table S1. This study was approved by the Ethics Committee (CES-IPO-12-018) of the Portuguese Oncology Institute of Porto, Portugal. All procedures performed in tasks involving human participants were in accordance with the ethical standards of the institutional and/or national research committee and with the 1964 Helsinki declaration and its later amendments or comparable ethical standards.

### 4.3. Immunohistochemistry

Antigenic recovery was performed for 20 min with citrate buffer in a microwave. The immunohistochemistry protocol used is described in full in [64]. Slides were incubated for one hour with primary antibodies at room temperature (Table S2). Tissue of normal gastric mucosae and gastric adenocarcinoma were used as external positive controls in each run. Negative controls, consisting of omission of primary antibodies, were included per run.

The immunoexpression of each target was assessed separately for each TGCT component (i.e., independently for each histological component within mixed tumors). As the staining was overall diffuse in all cases, but with great variation in staining intensity, we focused on the latter. The intensity of staining was considered as “weak”, “moderate”, and “strong”, as previously defined [65].

### 4.4. Cell Lines and Drugs

The (T)GCT cell lines (*n* = 4) TCam-2 (a SE-like cell line) and NCCIT, 2102Ep, and NT2 (representative of NS) were kindly provided by Prof. Leendert Looijenga. These cell lines have been previously characterized, including copy number alterations, and were cultured as described [66]. Additionally, an independent set of matched clones of cisplatin-sensitive and cisplatin-resistant cell lines (*n* = 6) were kindly provided by Prof. Daniel Nettersheim and generated by Dr. Christoph Oing and Prof. Friedemann Honecker. The resistant clones (NCCIT-R, 2102Ep-R, and NT2-R) were obtained from the parental matched clone (NCCIT-P, 2102Ep-P, and NT2-P) upon culturing with increasing doses of cisplatin, as reported before [67]. Cells were maintained in low passages and were negative for *Mycoplasma spp.* (Clontech Laboratories; Mountain View, CA, USA; tested twice a month). Cisplatin was kindly provided by IPO Porto’s Department of Pharmacy. Belinostat and panobinostat were purchased from Selleckchem, Houston, TX, USA (Catalog No. S1085 and Catalog No. S1030, respectively).

### 4.5. RNA Extraction, cDNA Synthesis, and RT-qPCR

Total RNA was extracted using FFPE RNA/DNA Purification Plus Kit (Cat. 54300, Norgen Biotek, Thorold, Canada) for FFPE tumor samples and TRIzol (Invitrogen, CA, USA) for cell lines. RNA quantification and purity were assessed in NanoDrop^TM^ Lite Spectrophotometer (Cat. ND-LITE, Thermo Scientific^TM^, MA, USA). cDNA synthesis (1000 ng) was performed as described elsewhere [64].

RT-qPCR was run in the LightCycler^®^ 480 multiwell plate system (Product no. 05015243001, Roche, Mannheim, Germany) using the following gene expression assays: *HDAC1, HDAC2, HDAC7, HDAC8, HDAC9,* and *HDAC11* (assay ID #10031226, BIORAD, PT). For normalization purposes, the 18S rRNA was used as housekeeping (TaqMan^TM^ gene expression, assay ID Hs99999901, Applied biosystems^®^, MA, USA). Results were plotted as relative expression of targets (target gene mean quantity/18S mean quantity), multiplied by 1000 for easier tabulation. Serial dilutions of cDNA obtained from Human Reference Total RNA (Cat. 750500, Agilent Technologies^®^, CA, USA) were used to compute standard curves for each plate. All experiments were run in triplicates and two negative controls were used in each plate. For cell lines, data was plotted using the 2^−ΔΔCt^ method. Five biological replicates were used, and reactions were also run in triplicates.

### 4.6. Protein Extraction and Quantification

Total protein was extracted from cells, in biological triplicates, using the radioimmunoprecipitation assay buffer (RIPA) (Santa Cruz Biotechnology Inc., Santa Cruz, TX, USA) complemented with 10% of protein inhibitor cocktail (PIC). After 15 min on ice, samples were centrifuged at 13,000 rpm for 30 min at 4 °C and the supernatant was collected. Protein was quantified using the Pierce BCA Protein Assay Kit (Thermo Scientific Inc., Waltham, MA, USA), according to manufacturer’s instructions.

### 4.7. Western Blot

Aliquots of 30 µg total protein from each cell line were resuspended in loading buffer, denatured at 95 °C for 5 min, and loaded in 8% or 12.5% polyacrylamide gels (as appropriate), where they were separated by size through sodium dodecyl sulfate-polyacrylamide gel electrophoresis (SDS-PAGE) at 120 V. Then, proteins were transferred to 0.2 μm polyvinylidene fluoride (PVDF) membranes (Bio-Rad Laboratories Inc., Hercules, CA, USA) using 25 mM Tris-base/glycine buffer and a Trans-Blot Turbo Transfer System (Bio-Rad) at 25 V and 1.3 mA for 10 to 15 min, as appropriate. Membranes were blocked with 5% bovine serum albumin (BSA; Santa Cruz, CA, USA) or 5% dry milk in TBS with 0.1% Tween (TBS-T, pH = 7.6) as appropriate, and then incubated with primary antibody (Table S2). Lastly, membranes were incubated with a secondary antibody coupled with horseradish peroxidase (Bio-Rad Laboratories Inc., Hercules, CA, USA), for 1 h at room temperature. To ascertain equal loading of protein, the membranes were incubated with an endogenous control antibody. Quantification was performed using band densitometry analysis from ImageJ software (version 1.6.1, National Institutes of Health, Bethesda, MD, USA), by comparing the specific protein band intensity with the loading control beta-actin (β-ACT). All quantifications were done in triplicates.

### 4.8. Immunofluorescence

Immunofluorescence for Ki67 was performed at 72 h of treatment with belinostat and panobinostat. Cells were seeded in 96-well plates at 6000 cells/well (seeding determined after optimization) and allowed to adhere at 37 °C, 5% CO_2_ overnight. On the next day, cells were given the respective treatments. At 72 h of exposure to the drugs, cells were fixed with 4% paraformaldehyde (PFA) for 10 min and permeabilized with 0.25% Triton X-100 solution in phosphate-buffered saline (PBS) for 15 min. Cells were then blocked with 5% BSA for 30 min, followed by incubation with Ki67 primary antibody (Table S2), overnight at room temperature. Cells were incubated with secondary antibody anti-mouse IgG- fluorescein isothiocyanate (FITC goat SLB4878, Sigma-Aldrich^TM^) for 1 h, at room temperature. Nuclear staining was performed with 4′,6- diamidino-2-phenylindole (DAPI; AR1176, BOSTER Biological Technologies, China) in mounting medium. The overall proportion of stained cells was ascertained in a fluorescence microscope. Pictures were taken by the fluorescence microscope Olympus IX51 with a digital camera Olympus XM10 using CellSens software (Olympus, Tokyo, Japan).

### 4.9. Cell Viability Assays

Briefly, the viability assay was performed at 24, 48, and 72 h of treatment with belinostat and panobinostat. Cells pre-treated with non-toxic concentrations of belinostat for 72 h were subsequently exposed to cisplatin for an additional 72 h (see more experimental details below). Cells were plated into 96-well plates in medium at a density of 6000 cells/well (seeding density previously optimized) and incubated overnight, at 37 °C in 5% CO_2_. In each experiment, the vehicle alone was included (dimethyl sulfoxide—DMSO—for HDACis, PBS for cisplatin).

For the viability assay, resazurin (Canvax Biotech, Córdoba, Spain) was used. The culture medium was removed, and cells were incubated for 3 h at 37 °C with 100 μL of 1:10 resazurin solution in culture medium. The solution was then removed, and spectrophotometric measurement was done at 560 nm (reference wavelength: 600 nm) in a microplate reader (Fluostar Omega, BMG Labtech, Germany). Wells with the resazurin solution were used as blank to correct the OD values. ODs obtained for each time point were all normalized for the 0-htime point. At each time-point, the HDACi and vehicle were freshly added to the wells and the procedure was repeated the next day. All experiments were performed with biological triplicates, each with experimental triplicates. IC50 values were extrapolated from the sigmoidal dose-response (four-parametric logistic equation) with a variable slope, as calculated on GraphPad Prism 6.

### 4.10. Proliferation Assays

The BrdU assay was performed at 24, 48, and 72 h of treatment with belinostat and panobinostat. Cells were plated into 96-well plates in medium at a density of 6000 cells/well (seeding density previously optimized) and incubated overnight at 37 °C in 5% CO_2_. At each time point, cells were previously incubated with 20 μM BrdU labeling solution for 12 h. After removing labeling medium, cells were fixed for 30 min at room temperature with FixDenat solution, after which anti-BrdU-POD antibody (1:100) was added. After 90 min the antibody was removed, and cells were rinsed three times with 1 × PBS. The immune complex formed was detected by adding 100 μL/well of substrate solution and incubated for 5 to 10 min, until color development. Then, the reaction was stopped with 1M H_2_SO_4_ added to each well, and the reaction product was quantified in a microplate reader by measuring absorbance at 450 nm (reference wavelength: 690 nm). ODs obtained for each time point were normalized for the 0-h time point. In each experiment, the vehicle alone was included. All experiments were performed with biological triplicates, each with experimental triplicates.

### 4.11. Apoptosis Assays

The apoptosis assay was performed at 24 h into treatment with belinostat and panobinostat. Cells were seeded in a 24-well plate at a density of 45,000 cells/well (seeding density determined after optimization) and incubated for 24 h at 37 °C and 5% CO_2_. After 24 h, the Cell-APOPercentage^TM^ apoptosis assay kit (Biocolor, Carrickfergus, United Kingdom) was followed according to the manufacturer’s instructions. Next, cells were incubated with 300 μL/well of APOPercentage^TM^ dye solution at a ratio of 1:20, for 30 min at 37 °C. Cells were washed with PBS 1× and detached from the wells with TrypLe^TM^ Express (GIBCO, Invitrogen, CA, USA) at 37 °C. Then, APOPercentage^TM^ dye release reagent was added and the plate was vigorously agitated for 15 min, followed by colorimetric measurement at 550 nm with 620 nm reference filter (Fluostar Omega, BMG Labtech, Offenburg, Germany). H_2_O_2_ was used as a positive control. ODs obtained were normalized for the 0-h time point. In each experiment, the vehicle alone was included (DMSO).

### 4.12. Statistical Analysis

Data were tabulated using Microsoft Excel 2016 and analyzed and plotted using GraphPad Prism 6. Percentages were calculated based on the number of cases with available data. Non-parametric (Mann–Whitney and Kruskal–Wallis) tests were used for comparing mRNA/protein expression levels among all samples (patients and cell lines), as necessary. All *p*-values were adjusted for multiple comparisons (Dunn’s test and Bonferroni correction, as appropriate). Chi-square and Fisher exact tests were used as necessary for establishing associations between categorical variables. Statistical significance was set at *p* < 0.05 and is reported in graphs as following: * *p* < 0.05; ** *p* < 0.01; *** *p* < 0.001; **** *p* < 0.0001.

## 5. Conclusions

To conclude, we demonstrated, for the first time, the effectiveness of two recently FDA-approved agents, belinostat and panobinostat, for treating cisplatin-sensitive and, importantly, cisplatin-resistant (T)GCT cell lines, reducing cell viability, inducing cell cycle arrest and apoptosis. HDACis show promise as single or combination agents, and our work builds up evidence of their effectiveness for treating TGCT patients developing cisplatin resistance, which, in our view, should motivate clinical trials for assessing the clinical utility of these epidrugs.

## Supplementary Materials

The following are available online at https://www.mdpi.com/2072-6694/12/10/2903/s1, Figure S1: Transcript levels of several HDACs across individual TGCT subtypes, assessed in our patient cohort. (A) HDAC1, (B) HDAC2, (C) HDAC7, (D) HDAC8, (E) HDAC9, (F) HDAC11. (G) Differential expression of HDAC7 across disease stage. Bars and red dashes represent median and interquartile range. Abbreviations: HDAC—histone deacetylase; SE—seminoma; YST—yolk sac tumor; CH—choriocarcinoma; TE—teratoma; EC—embryonal carcinoma, Figure S2: Differential transcript and protein levels of several HDACs across (T)GCT cell lines NCCIT, NT2, 2102Ep, and TCam-2. (A–F) mRNA expression; (G–I) protein expression, Figure S3: Demonstration of cisplatin resistance and sensitivity in the cell lines used in the study. Cell viability after 72 h of exposure to cisplatin in NCCIT-R (A), NCCIT-P (B), 2102Ep-R (C), 2102Ep-P (D), NT2-R (E), and NT2-P (F), Figure S4: Barplots with cell viability studies after treatment with several doses of belinostat and panobinostat, per time point (24, 48, and 72 h), across cisplatin-resistant cell lines. (A) NCCIT-R; (B) 2102Ep-R; (C) NT2-R, Figure S5: Barplots with cell viability studies after treatment with several doses of belinostat and panobinostat, per time point (24, 48, and 72 h), across cisplatin-sensitive cell lines. (A) NCCIT-P; (B) 2102Ep-P; (C) NT2-P, Figure S6: Effect of treatment of the NCCIT-R cell line with belinostat and panobinostat on cell cycle, apoptosis, and acetylation. (A) Effect of treatment with belinostat (50 and 100 nM) and with panobinostat (5 and 10 nM) for 72 h on Ki67 staining index; (B) Western blot validation of specific targets related to cell cycle (p21, p53), apoptosis (cleaved caspase 3) and acetylation (lysine acetylation, histone H3 acetylation, HDAC1) after treatment for 24 h with belinostat and panobinostat. Experiments were performed in triplicates. Beta-actin is presented as normalizer, Figure S7: Effect of pre-treatment with non-toxic low nanomolar concentrations of belinostat on sensitivity to cisplatin. (A) Timeline of the experiment, with daily belinostat treatments for three days vs absence of treatment, followed by exposure to cisplatin 10 µM for 72 h; (B) Respective viability curves across the time of the experiment. Abbreviations: DMSO—dimethyl sulfoxide, Table S1: Clinicopathological features of the study cohort, Table S2: Antibodies used in the study.
