# Supplementary material for: Efficacy of HDAC Inhibitors Belinostat and Panobinostat against Cisplatin-Sensitive and Cisplatin-Resistant Testicular Germ Cell Tumors
Source: Cancers (Basel). 2020 Oct 10;12(10):2903. doi: 10.3390/cancers12102903 (PMC7601457; doi:10.3390/cancers12102903)
Supplement: Supplementary file 1 [file cancers-12-02903-s001.pdf]

cancers MDPI

Supplementary Materials: Efficacy of HDAC Inhibitors Belinostat and Panobinostat against Cisplatin-Sensitive and Cisplatin-Resistant Testicular Germ Cell Tumors

João Lobo, Catarina Guimarães-Teixeira, Daniela Barros-Silva, Vera Miranda-Gonçalves, Vânia Camilo, Rita Guimarães, Mariana Cantante, Isaac Braga, Joaquina Maurício, Christoph Oing, Friedemann Honecker, Daniel Nettersheim, Leendert HJ Looijenga, Rui Henrique and Carmen Jerónimo

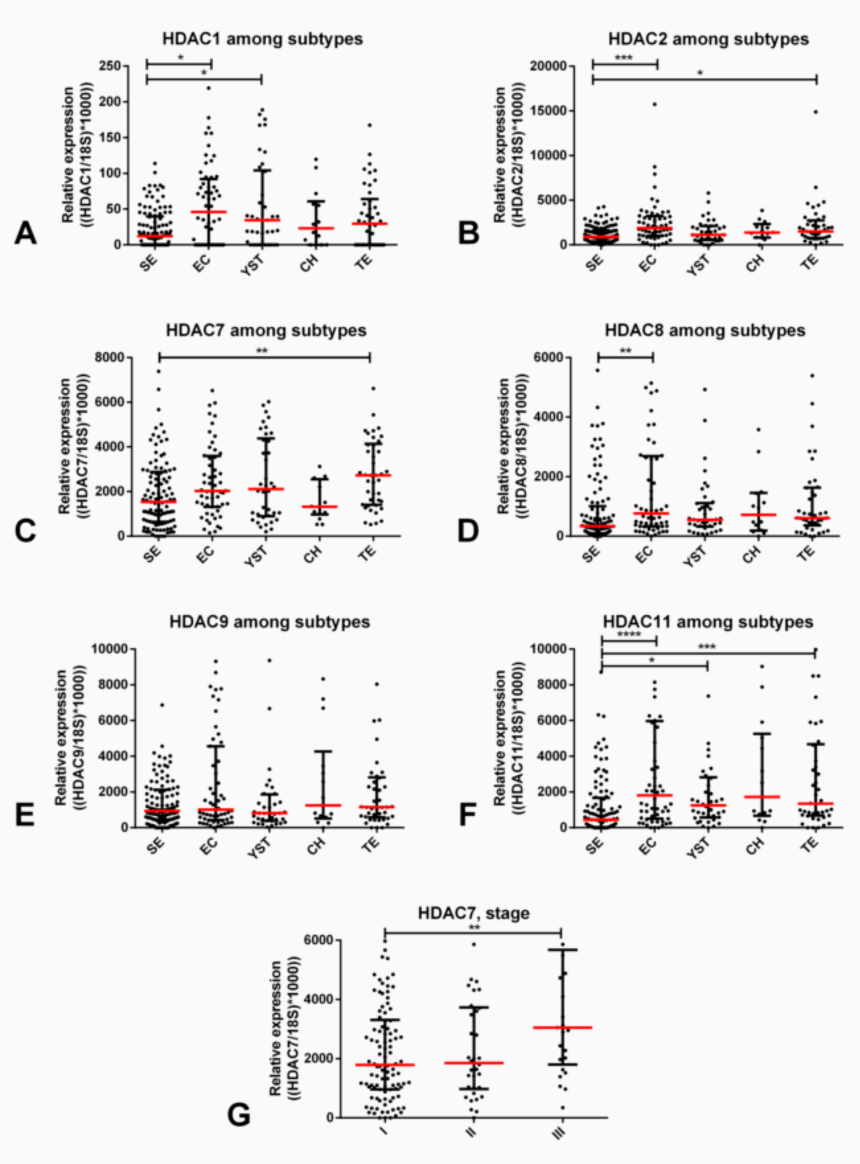

Figure S1. Transcript levels of several HDACs across individual TGCT subtypes, assessed in our patient cohort. (**A**) HDAC1, (**B**) HDAC2, (**C**) HDAC7, (**D**) HDAC8, (**E**) HDAC9, (**F**) HDAC11. (**G**) Differential expression of HDAC7 across disease stage. Bars and red dashes represent median and interquartile range. Abbreviations: HDAC—histone deacetylase; SE—seminoma; YST—yolk sac tumor; CH—choriocarcinoma; TE—teratoma; EC—embryonal carcinoma. .*?; **?; ***?, ****?

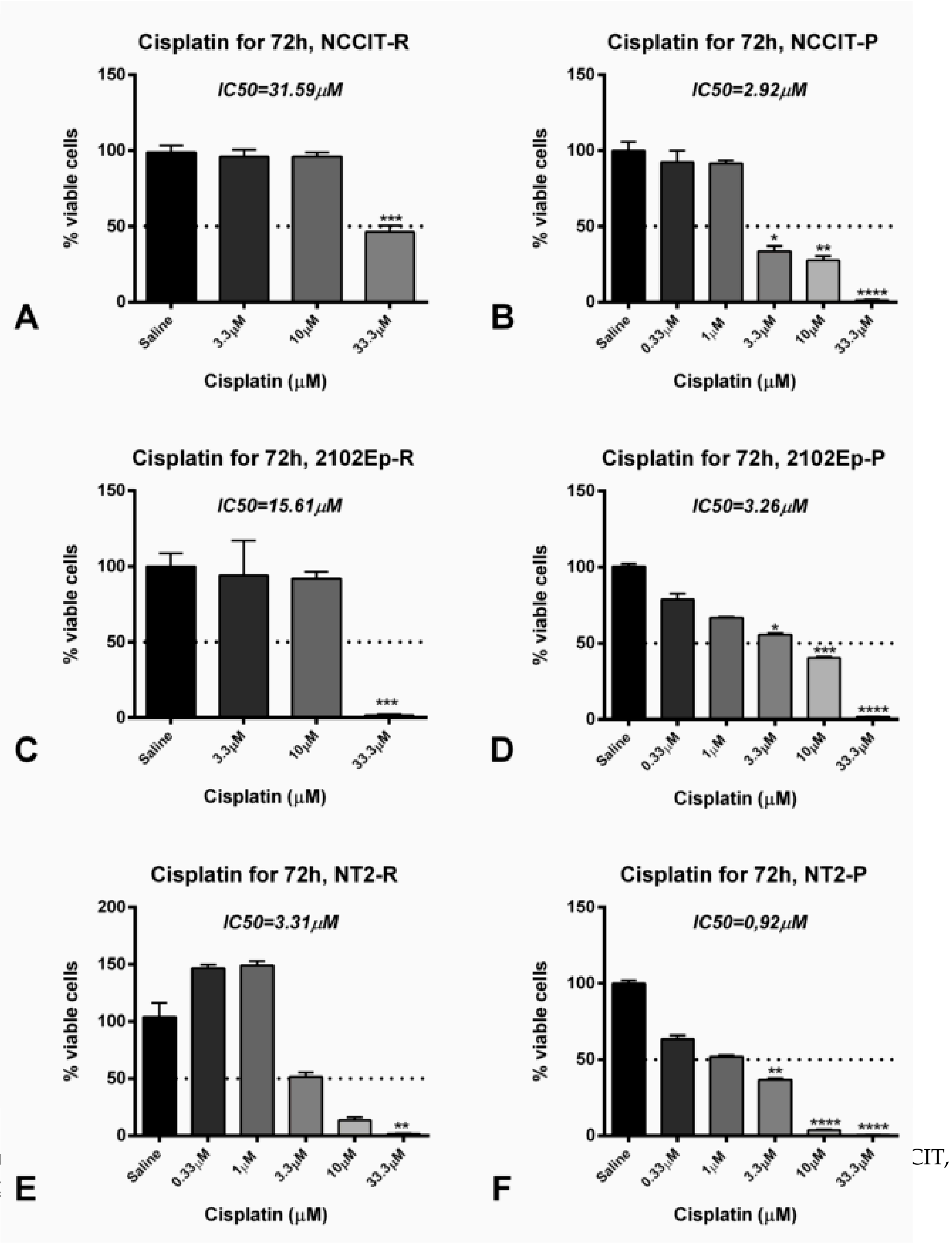

Fig[illegible] [illegible]CIT,
NT2[illegible]

Figure S3. Demonstration of cisplatin resistance and sensitivity in the cell lines used in the study. Cell viability after 72 h of exposure to cisplatin in NCCIT-R (**A**), NCCIT-P (**B**), 2102Ep-R (**C**), 2102Ep-P (**D**), NT2-R (**E**), and NT2-P (**F**). .*?; **?; ***?, ****?

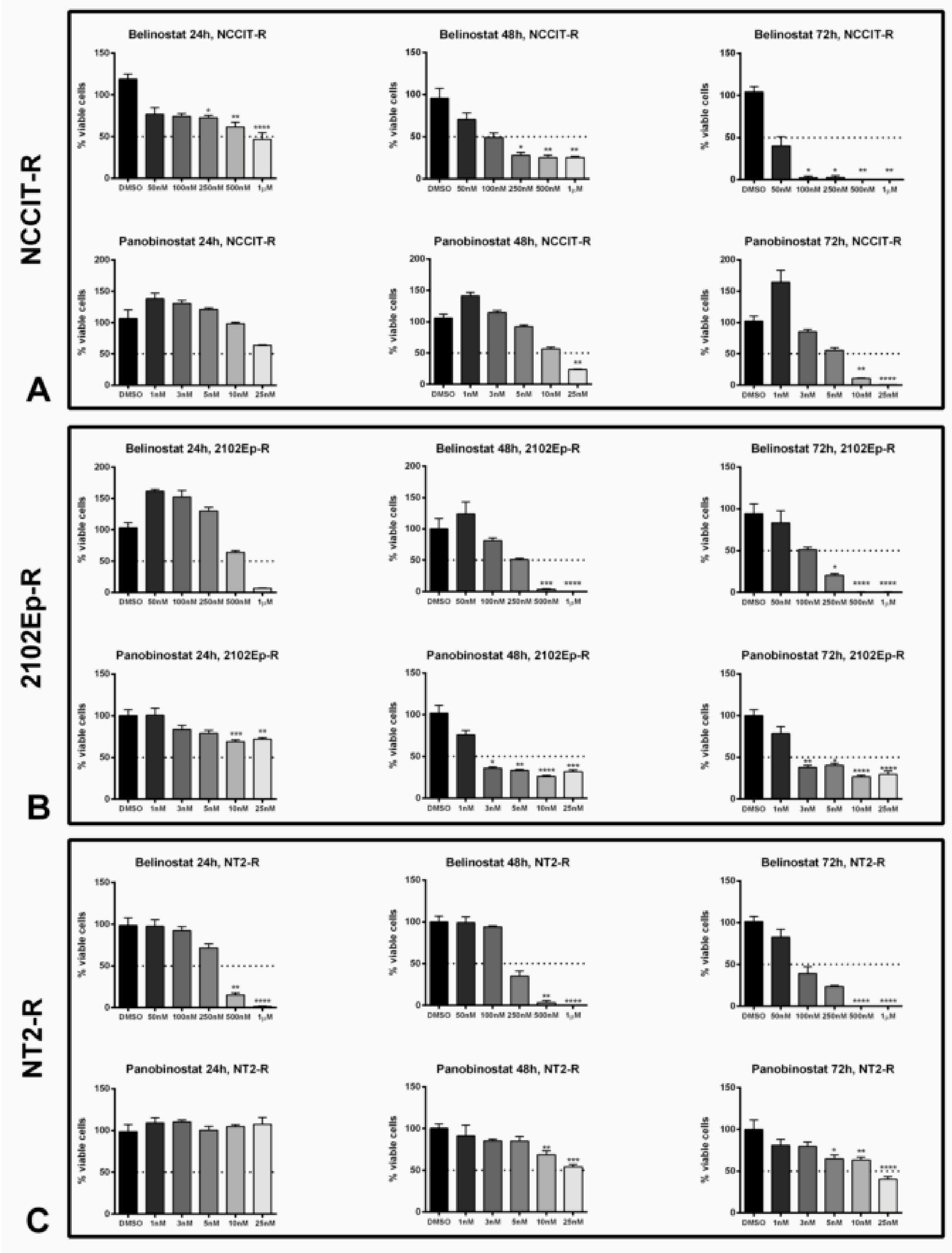

Figure S4. Barplots with cell viability studies after treatment with several doses of belinostat and panobinostat, per time point (24, 48, and 72 h), across cisplatin-resistant cell lines. (**A**) NCCIT-R; (**B**) 2102Ep-R; (**C**) NT2-R. .*?; **?; ***?, ****?

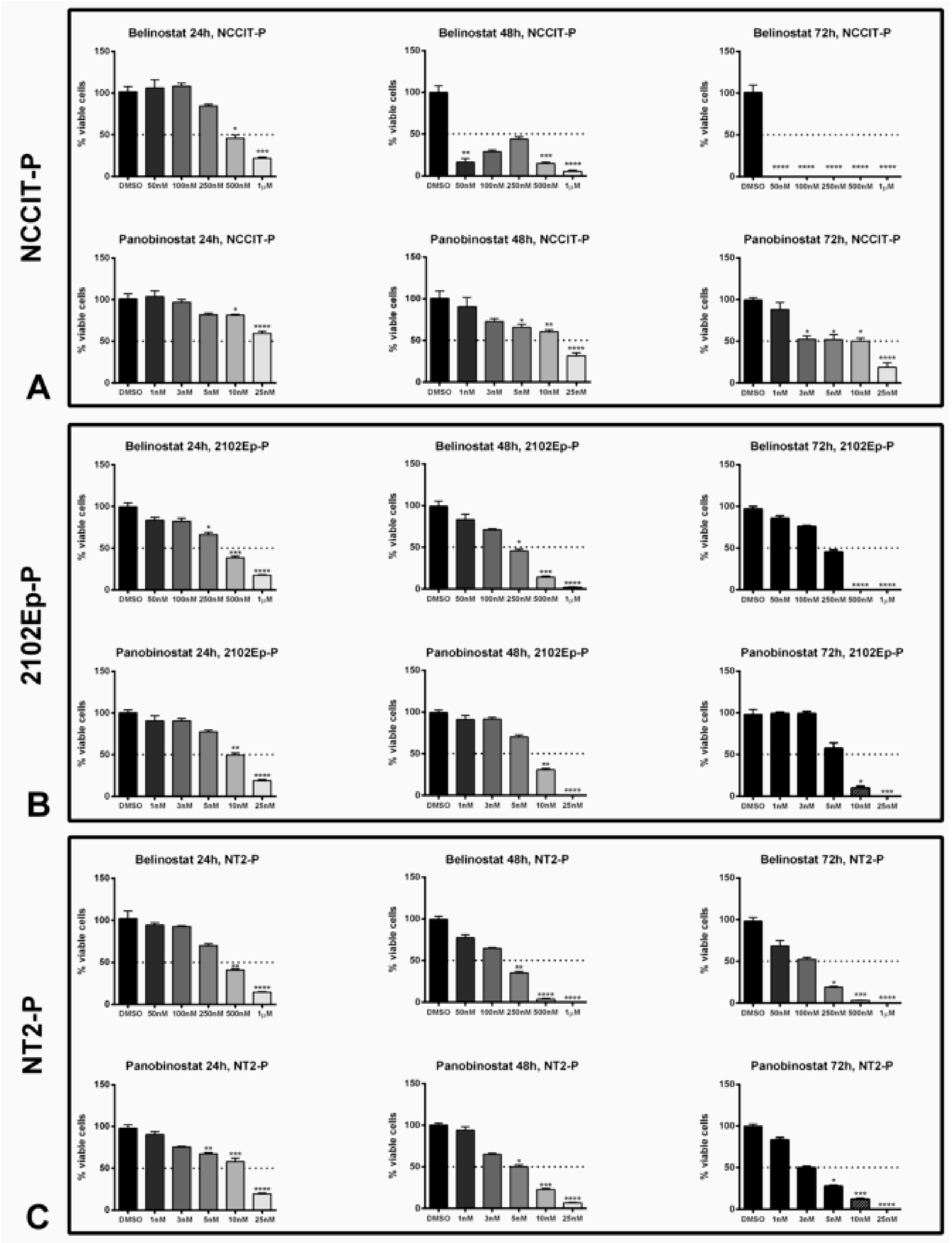

Figure S5. Barplots with cell viability studies after treatment with several doses of belinostat and panobinostat, per time point (24, 48, and 72 h), across cisplatin-sensitive cell lines. (**A**) NCCIT-P; (**B**) 2102Ep-P; (**C**) NT2-P. .*?; **?; ***?, ****?

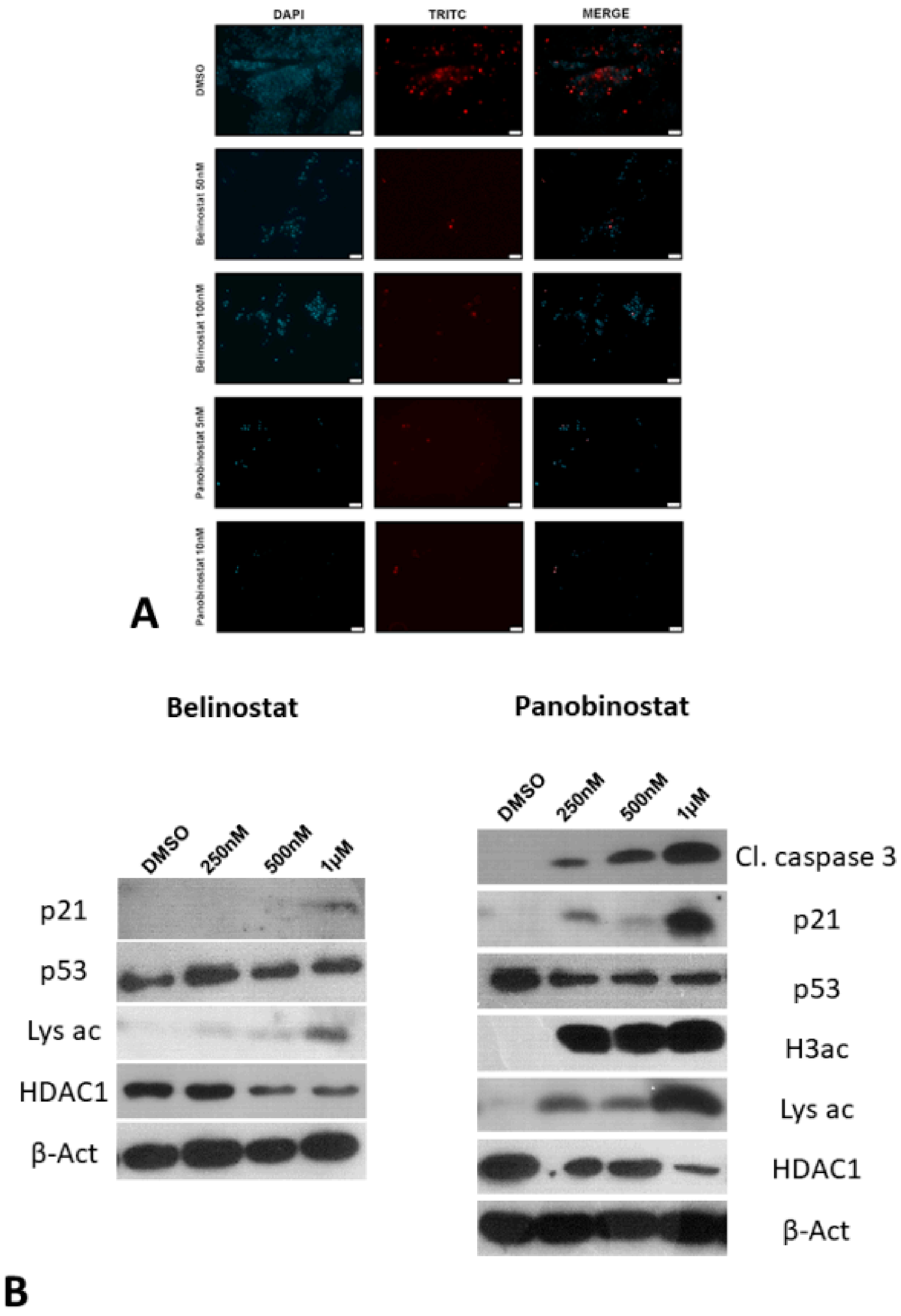

Figure S6. Effect of treatment of the NCCIT-R cell line with belinostat and panobinostat on cell cycle, apoptosis, and acetylation. (**A**) Effect of treatment with belinostat (50 and 100 nM) and with panobinostat (5 and 10 nM) for 72 h on Ki67 staining index; (**B**) Western blot validation of specific targets related to cell cycle (p21, p53), apoptosis (cleaved caspase 3) and acetylation (lysine acetylation, histone H3 acetylation, HDAC1) after treatment for 24 h with belinostat and panobinostat. Experiments were performed in triplicates. Beta-actin is presented as normalizer. scale bars??

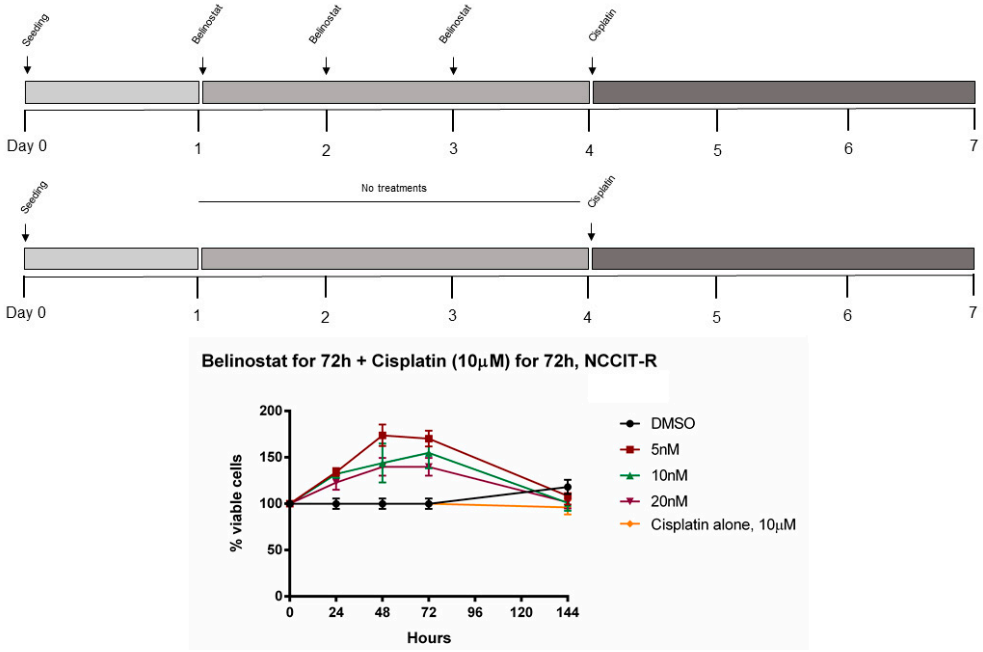

Figure S7. Effect of pre-treatment with non-toxic low nanomolar concentrations of belinostat on sensitivity to cisplatin. (**A**) Timeline of the experiment, with daily belinostat treatments for three days vs absence of treatment, followed by exposure to cisplatin 10 μM for 72 h; (**B**) Respective viability curves across the time of the experiment. Abbreviations: DMSO—dimethyl sulfoxide.

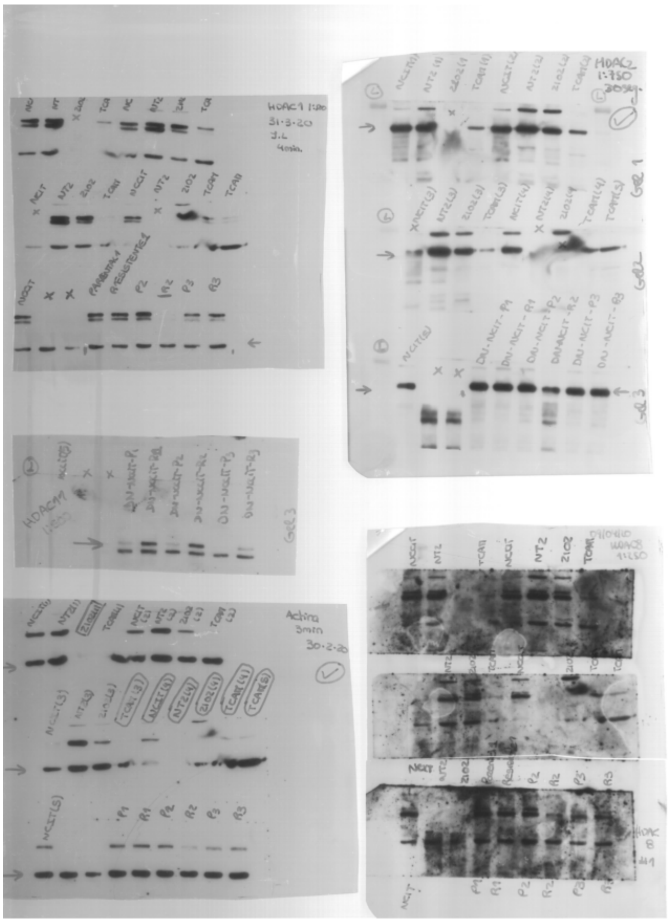

Figure S8

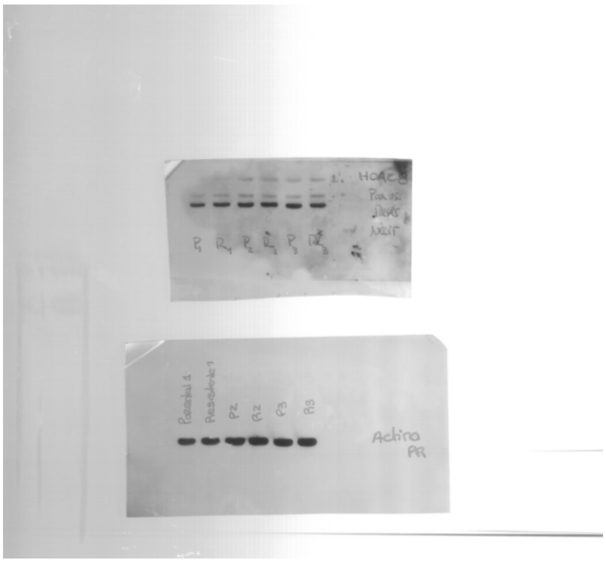

Figure S9

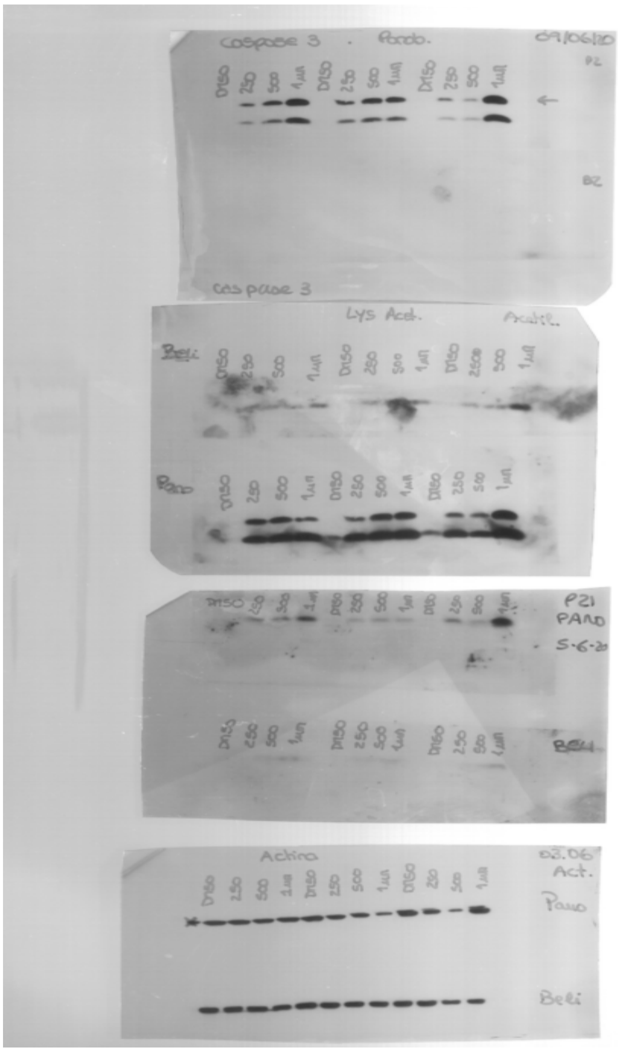

Figure S10

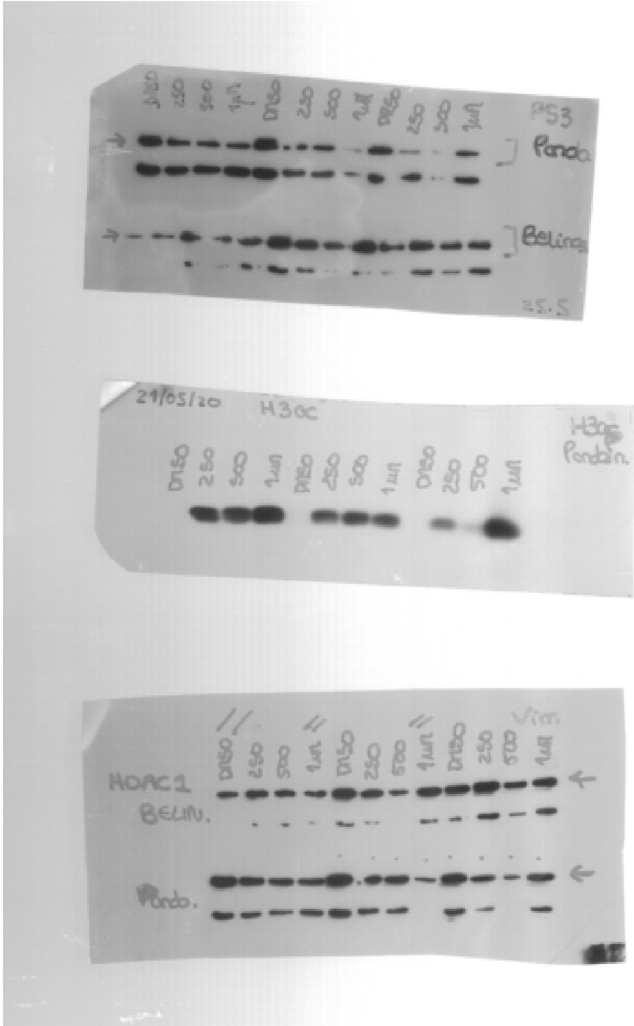

Figure S11

Table S1. Clinicopathological features of the study cohort.

Variables	Primary TGCT Cases (*n*, %)
Histologic subtypes – TGCT patients (*n*, %)	
Pure seminoma	84/161 (52.2)
Pure embryonal carcinoma	11/161 (6.8)
Pure postpubertal-type teratoma	4/161 (2.5)
Mixed tumor	62/161 (38.5)
Histological subtypes – individual components (*n*, %)	
Seminoma	109/261 (41.8)
Embryonal carcinoma	56/261 (21.5)
Postpubertal-type yolk sac tumor	38/261 (14.5)
Choriocarcinoma	15/261 (5.7)
Postpubertal-type teratoma	43/261 (16.5)
Stage (*n*, %)	
I	102/161 (63.4)
II	34/161 (21.1)
III	25/161 (15.5)
IGCCCG Prognostic Group, for metastatic patients (*n*, %)	
Good	45/59 (73.8)
Intermediate	8/59 (14.3)
Poor	6/59 (11.9)
Variables	Metastatic cases (*n*, %)

Histologic subtypes (*n*, %)	
Seminoma	1/14 (7.1)
Embryonal carcinoma	3/14 (21.5)
Yolk sac tumor	2/14 (14.3)
Teratoma	8/14 (57.1)

Abbreviations: TGCT–testicular germ cell tumors; IGCCCG–International Germ Cell Cancer Collaborative Group.

Table S2. Antibodies used in the study.

Antibody	Clone/Ref, Species	Vendor	Dilution
HDAC1	5C11, mouse	Sigma-Aldrich	1:500
HDAC2	HDAC2-62, mouse	Sigma-Aldrich	1:750
HDAC8	2F4, mouse	Novus-Biologicals	1:250
HDAC11	D5I8E, rabbit	Cell Signaling	1:500
Acetylated lysine	#9441, rabbit	Cell Signaling	1:500
H3ac	Polyclonal, rabbit	Merk, millipore	1:1000
p53	OP43, mouse	Oncogene science	1:1000
p21	SX118, mouse	Pharmingen	1:250
Ki67	MIB-1, mouse	DAKO	1:200
Cleaved caspase 3	Polyclonal, rabbit	Abcam	1:500
β-actin	AC-15, mouse	Sigma-Aldrich	1:10000

© 2020 by the author. Licensee MDPI, Basel, Switzerland. This article is an open access article distributed under the terms and conditions of the Creative Commons Attribution (CC BY) license (http://creativecommons.org/licenses/by/4.0/).
